# Advancing Atrial Fibrillation Screening—Clinical Utility and Future Directions of Smartwatch ECG

**DOI:** 10.1002/joa3.70143

**Published:** 2025-07-16

**Authors:** Keitaro Senoo

**Affiliations:** ^1^ Department of Cardiac Arrhythmia Research and Innovation Graduate School of Medical Science, Kyoto Prefectural University of Medicine Kyoto Japan; ^2^ Department of Cardiovascular Medicine Graduate School of Medical Science, Kyoto Prefectural University of Medicine Kyoto Japan

**Keywords:** atrial fibrillation, electrocardiogram, smartwatch

Atrial fibrillation (AF) continues to rise globally and is well known to increase the risk of serious cardiovascular complications, such as ischemic stroke and thromboembolism. With the advent of an aging society, early detection and prevention of AF have become urgent challenges, not only for individual health but also from a healthcare economics perspective. In this context, smartwatch‐based single‐lead electrocardiograms (ECG) have attracted attention as a noninvasive and rapid method for acquiring ECG data during daily life.

The present study, “Accuracy and Interpretability of Smartwatch Electrocardiogram for Early Detection of Atrial Fibrillation,” [1] focuses on the accuracy and interpretability of smartwatch ECG in detecting AF and evaluates its effectiveness through a quantitative meta‐analysis. The authors systematically reviewed literature indexed in major databases, including Scopus, PubMed, and Web of Science, and performed meta‐analyses using a two‐level mixed‐effects logistic regression model and a Freeman‐Tukey double arcsine transformation.

The findings revealed promising results: algorithm‐based automatic readings demonstrated a sensitivity of 86% and a specificity of 94%, while manual readings by healthcare professionals achieved even higher sensitivity and specificity of 96% and 95%, respectively. Notably, devices such as the Withings Scanwatch and Apple Watch showed particularly high clinical reliability, with summary area under the curve (sAUC) values of 96% and 98%, respectively. Furthermore, the interrater agreement for manual interpretation was substantial (Cohen's kappa = 0.83), with only 3% of ECG tracings deemed uninterpretable.

The significance of this study lies in its systematic and quantitative demonstration of the high diagnostic accuracy of smartwatch ECG in AF screening. Particularly in high‐risk populations, a two‐step approach—initial screening using smartwatch ECG followed by clinical confirmation for positive cases—presents a realistic and efficient strategy.

From the editorial perspective, we propose such a practical approach that may enhance the effectiveness of AF screening, particularly in high‐risk populations. In this suggested workflow, the first step involves individuals recording ECGs through their smartwatches during routine self‐monitoring. If the built‐in algorithm detects a possible AF episode, a notification is issued. In the second step, the ECG data could be transmitted to a remote physician review service, where a clinical expert re‐evaluates the tracing. Based on this review, triage decisions—such as “high likelihood of AF, recommend clinical consultation,” “unclear findings, suggest further testing,” or “no abnormality, continue observation”—can be made. This strategy may reduce unnecessary in‐person visits while ensuring that those at risk receive timely and appropriate care. Such a two‐step approach “a system comprising smartwatch ECG, remote review, and clinical triage” represents a highly practical and efficient system. Integration with electronic health records enables real‐time data access and accelerates clinical decision‐making, offering a significant advantage in today's healthcare landscape.

Looking ahead, this model of remote screening and review may eventually be eligible for public insurance coverage in various healthcare systems. As societies face growing pressure to manage healthcare costs in aging populations, high‐accuracy, noninvasive screening tools such as smartwatch ECGs may receive increasing institutional recognition. Although the regulatory and reimbursement pathways vary by country, demonstrating cost‐effectiveness and clinical utility will be key to broader adoption.

Of course, this technology has limitations, such as motion artifacts and restrictions on the heart rate range. Motion artifacts refer to the noise generated when the hands or body move while wearing the smartwatch, which can distort the ECG waveform and make accurate analysis difficult. Additionally, most current smartwatch ECGs are designed with the assumption that the heart rate falls within a certain range (e.g., 50 to 150 beats per minute on the Apple Watch, 50 to 120 beats per minute on the Samsung Galaxy Watch, 50 to 100 beats per minute on the Withings Scanwatch, etc.). In cases of extreme bradycardia or tachycardia outside of this range, the sensitivity for detecting AF may decrease. However, by understanding these technical limitations and using complementary methods, the more effective use of smartwatch ECG is anticipated.

While further studies are warranted to validate the real‐world effectiveness and cost‐efficiency of smartwatch‐based ECG screening, the present meta‐analysis provides compelling evidence supporting its potential as a powerful tool for early detection of AF. Real‐world effectiveness should be demonstrated not only through improved detection rates—especially in asymptomatic or high‐risk populations—but also by showing tangible clinical benefits such as reduced incidence of stroke, timely initiation of anticoagulation therapy, and lowered rates of hospitalization. Moreover, seamless integration into routine clinical workflows, enhanced patient adherence, and satisfaction with remote monitoring platforms would further underscore its practical value. From a cost‐efficiency perspective, a two‐tiered approach involving algorithm‐based preliminary screening followed by remote expert review may optimize healthcare resource allocation, reduce unnecessary consultations, and ensure timely interventions for those truly in need. Such a strategy holds promise in aging societies where health systems face increasing demands and limited resources.

In conclusion, this study illustrates the transformative potential of integrating wearable technology with clinical decision‐making, marking a significant step forward in preventive cardiology and personalized healthcare delivery.

## Disclosure

The author has nothing to report.

## Conflicts of Interest

The author declares no conflicts of interest.

## References

[1] “Accuracy and Interpretability of Smartwatch Electrocardiogram for Early Detection of Atrial Fibrillation: A Systematic Review and Meta‐Analysis,” Journal of Arrhythmia 41, no. 3 (2025): e70087. 40406413 10.1002/joa3.70087 PMC12096014

